# The Role of Animal Ownership for People with Severe Mental Illness during the COVID-19 Pandemic: A Mixed-Method Study Investigating Links with Health and Loneliness

**DOI:** 10.3390/ijerph182211908

**Published:** 2021-11-12

**Authors:** Emily Shoesmith, Panagiotis Spanakis, Emily Peckham, Paul Heron, Gordon Johnston, Lauren Walker, Suzanne Crosland, Elena Ratschen

**Affiliations:** 1 Department of Health Sciences, University of York, York YO10 5DD, UK; panagiotis.spanakis@york.ac.uk (P.S.); emily.peckham@york.ac.uk (E.P.); paul.heron@york.ac.uk (P.H.); lauren.walker@york.ac.uk (L.W.); suzanne.crosland@york.ac.uk (S.C.); elena.ratschen@york.ac.uk (E.R.); 2 Independent Researcher, Clackmannan FK10 4EF, UK; g_johnston@btinternet.com

**Keywords:** human–animal interaction, human–animal relationships, companion animals, COVID-19, mental health, physical health, well-being, loneliness, severe mental illness

## Abstract

Research has reported the benefits of companion animals for people with severe mental illness (SMI). However, this evidence base is fragmented and unclear. The COVID-19 pandemic presents an opportunity to explore the role of companion animals in the context of social distancing and isolation measures for people with SMI. Therefore, we aimed to investigate the links between mental and physical health and animal ownership in people with SMI and to explore animal owners’ perceptions related to human–animal interactions during the pandemic restrictions. A survey was conducted with a previously assembled cohort of individuals with SMI in the UK. The survey included previously validated and new bespoke items measuring demographics, and outcomes related to mental and physical health, and human–animal interactions. The survey also included a question inviting free-text responses, allowing participants to describe any experiences of their human–animal relationships during the pandemic. Of 315 participants who consented to participate, 249 (79%) completed the survey. Of these, 115 (46.2%) had at least one companion animal. Regression analyses indicated that animal ownership was not significantly associated with well-being and loneliness. However, animal ownership was associated with a self-reported decline in mental health (*b* = 0.640, 95% CI [0.102–1.231], *p* = 0.025), but no self-reported change in physical health. Thematic analysis identified two main themes relating to the positive and negative impact of animal ownership during pandemic restrictions. Animal ownership appeared to be linked to self-reported mental health decline in people with SMI during the second wave of the pandemic in the UK. However, the thematic analysis also highlighted the perceived benefit of animal ownership during this time. Further targeted investigation of the role of human–animal relationships and the perceived human–animal bond for human health is warranted.

## 1. Introduction

Human–animal interaction (HAI) is a broad term referring to various relationships or interactions between a human and an animal [1]. There has been increasing recognition of the ownership of companion animals and their potential impact on physical and mental health [2], particularly within the context of the COVID-19 pandemic [3,4].

An increasing number of studies report that owning a companion animal can be beneficial for mental health and well-being, for example, through hypothesised mechanisms involving attachment to or social support received from the animal [4,5]. Previous literature has highlighted the relevance of companion animals for the social networks of people who have received a diagnosis of SMI (e.g., schizophrenia and bipolar disorder) [6], suggesting that companion animals should usefully be considered in addition to human relationships. However, existing research investigating the benefit of animal ownership for those with diagnosed mental health conditions is unclear and fragmented [2]: There is evidence to illustrate that companion animal ownership can have a range of benefits for people with SMI, including facilitating the development of coping skills [6,7]; providing feelings of distraction and a form of activity encouragement [6], and supporting self-efficacy and enhancing one’s sense of empowerment [8]. Conversely, there is evidence that, rather than improving owners’ mental health or well-being, strong attachment to companion animals can be associated with worse outcomes for depression and loneliness, predicting vulnerability in owners [9,10]. Research has indicated that animal owners have a significantly higher likelihood of experiencing depression or anxiety [11,12,13], and it is possible that the responsibility of providing care for an animal is connected to negative mental health outcomes [14,15,16,17]. Thus, there is a consensus in this developing, interdisciplinary field that considerable scope for targeted research to investigate the relationships between humans and animals for health and well-being exists [5,9,10].

Since the beginning of the COVID-19 pandemic, research has emerged investigating human–animal relationships and interactions in the context of pandemic-related social restrictions. An increasing number of studies have explored human–animal interactions and the links with mental health within the pandemic context [3,4,18,19,20]. However, these findings may not replicate across other population groups, as existing studies have explored the role of companion animals in the general population [3,4,18,21], adolescents [19], children [22], and older adults [23]. There has been a dearth of literature investigating the role companion animals may play for people with SMI during the COVID-19 pandemic. Studies that have focused on this adult population outside of a pandemic context are often specific to some companion animal species (primarily dogs and cats) [24] or investigate the impact of animal-assisted interventions as opposed to the benefits or challenges of owning companion animals [25,26]. Therefore, using data from a larger, multi-domain survey, this study aimed to investigate the following research questions:

1. Are well-being and loneliness associated with animal ownership in people with SMI during the restrictions implemented during the second wave of the COVID-19 pandemic? (RQ1)
2. Is reported deterioration or improvement in physical and mental health associated with animal ownership and regular engagement with companion animals? (RQ2)
3. How do companion animal owners perceive the influence of human–animal interaction on their physical and mental health during the pandemic restrictions? (RQ3)

## 2. Materials and Methods

### 2.1. Study Design

A survey was completed online, via telephone, or post. This survey was part of a larger, two-part, mixed-method study exploring the effects of the pandemic restrictions on people with SMI. This study used an embedded mixed-methods design [27], as it is possible neither quantitative nor qualitative methods are sufficient to comprehend the association between animal ownership and health outcomes when used independently. Therefore, the purpose of using this approach was to obtain qualitative data that could support the interpretation of quantitative findings.

The current study reports findings from the follow-up survey. For a more detailed account of the methodology, see Supplementary Material S1. Items measuring outcomes related to human–animal interactions were included in the follow-up survey as participants who completed the first survey had indicated the importance of their companion animal during the COVID-19 pandemic. Therefore, it was considered important to understand more about the role of human–animal relationships and interactions for mental and physical health.

### 2.2. Settings and Participants

This survey was conducted within a previously assembled cohort (Closing the Gap: Health and Well-being Cohort; CtG) of nearly 10,000 people in the UK with SMI (defined here as schizophrenia or other psychotic disorders (ICD-10 [28] codes F20.x and F22.X or DSM IV or V [29] equivalent) or bipolar disorder (ICD-10 code F31.X or DSM equivalent). The composition of the CtG cohort has been described elsewhere [30].

We were funded to explore the impact of the COVID-19 pandemic in a sub-section of the CtG clinical cohort and participants were identified for Optimising Well-being in Self-Isolation Study (OWLS). To confirm that the OWLS COVID-19 sub-cohort included various demographics, a sampling framework based on gender, age, ethnicity, and recruitment via primary or secondary care, was created. OWLS participants were recruited from mental health trusts (n = 17) and clinical research networks (n = 6), across both rural and urban English settings.

To be eligible to take part, participants had to be aged 18 or over, previously participated in the CtG study, and consented to be contacted again to participate in future research following completion of the first OWLS survey.

### 2.3. Measures

A multi-disciplinary team of academics developed a bespoke questionnaire. An overview of the measures included in the current study is provided below.

Demographic data: Demographic information including participants’ age, gender (male, female, transgender), and ethnicity (e.g., White, Mixed, Black) were collected at the inception of the CtG cohort. An additional demographic question about their professional activity was included in the OWLS survey. Based on professional activity, participants were categorised as those professionally active (e.g., employed full time or part time, self-employed, volunteering, studying) or those not professionally active (e.g., retired, not in paid employment, not engaging in studying or volunteering).

Companion animal ownership: Participants were asked, ‘Do you have any animals that live with you or near you, and that you or anyone in your household are the main caretaker of? Please do not include animals kept as livestock (e.g., farm sheep, cattle)’. If answering ‘yes’, they were asked to indicate how many and which species (dog, cat, small mammal, bird, fish, reptile or amphibian, horse or pony, farm animal, other).

Non-animal owners were asked why they did not own animals (e.g., ‘I am not interested in owning an animal’; ‘I would like an animal, but my circumstances do not allow it’; ‘I have recently lost an animal and am not yet ready to have another one’). Participants were able to indicate agreement to multiple statements to describe why they did not own a companion animal.

Engagement with companion animals: Participants who owned companion animals were asked to identify the animal they felt closest to. With this animal in mind, participants were asked to indicate agreement to the following three items on a four-point Likert scale (1 = never; 4 = almost always): ‘do you spend time each day playing with or exercising your pet?’; ‘when you feel bad, do you seek your pet for comfort?’, and ‘how often do you consider your pet to be a member of your family?’.

General mental health and physical health: Participants were asked to rate 1) their mental health and 2) their physical health, compared with 6 months ago, by selecting one of the following responses: ‘better than before’; ‘about the same’; ‘worse than before’ ‘not sure’; ‘do not know’. The overall direction of the OWLS project was to understand what kept participants from declining in terms of self-perceived mental health since the start of the pandemic. Therefore, after excluding participants reporting ‘not sure’ and ‘do not know’, a binary variable was derived for analysis coded as ‘deterioration in health’ (including those reporting worse than before) or ‘no deterioration in health’ (including those reporting about the same or better than before). This categorisation remains across all OWLS projects for consistency.

Global health and well-being: Four questions were taken from the ONS Health and Lifestyle Survey (HLS) [31], asking participants to indicate how they had been feeling on a Likert scale of 0–10 (0 = not at all; 10 = completely) as follows: ‘overall, how satisfied are you with your life nowadays?’; ‘overall, to what extent do you feel that the things you accomplish in your life are worthwhile?’; ‘overall, how happy did you feel yesterday?’, and ‘overall, how anxious did you feel yesterday’. The score for the fourth item was reversed, and the total score for all items was calculated and treated as a continuous variable for analyses, with higher total scores indicating greater well-being.

Loneliness: The 3-item short version of the UCLA loneliness scale [32] was included. Participants were asked to indicate agreement to the three items on a 3-point Likert scale (1 = hardly ever; 3 = often) considering the last two weeks in their responses. Higher scores on this scale represent greater loneliness.

Free-text responses: Participants had the option to enter an open-ended, free-text comment at the end of the survey to describe their perceptions and experiences of any human–animal interactions or relationships during the second wave of pandemic restrictions in the UK. The item read, ‘Please let us know anything else you would like to tell us about what your animal(s) mean to you in the COVID-19 context or any other related subject you would wish to cover’.

### 2.4. Recruitment and Procedures

Participants who consented to follow-up in the first survey (OWLS 1) were contacted via telephone or post and invited to participate in the follow-up survey (OWLS 2). Those who agreed to participate were able to opt for their preferred method of completing the survey, which was to (1) complete the survey via telephone with a member of the research team, (2) complete the survey online via a link sent by a member of the research team, or (3) complete a hard copy of the survey and return via post. For those who completed the survey remotely, a Participant Information Sheet was provided via email, text message, or read to the participant via a telephone call. For those who completed a hard copy of the survey, a Participant Information Sheet was provided with the survey via post. Voluntary completion of the survey indicated consent to participate in the study.

OWLS 2 commenced during the second wave of the COVID-19 pandemic in January 2021, when strict social distancing and social isolation measures were implemented in the UK. Data collection ended in March 2021, when lockdown measures were easing, including gradual relaxing of social distancing rules.

Ethical approval for the survey was granted by the Northwest-Liverpool Central Research Ethics Committee (ref no. 20/NW/0276). All participants provided informed consent to take part.

### 2.5. Data Analysis

Descriptive statistics are provided for demographic information and data relating to animal ownership and non-ownership.

To address RQ1, we conducted a linear regression to assess the association of animal ownership and well-being score, controlling for covariates gender, age, ethnicity, and professional activity. Additionally, linear regression analyses were conducted to assess whether animal ownership was associated with the loneliness score, adjusting for the same covariates.

To investigate whether self-reported changes in mental health and physical health were associated with animal ownership and regular engagement with companion animals in animal owners (RQ2), separate binary logistic regression analyses were conducted. These assessed the association between each predictor (animal ownership: yes/no; spending time each day playing with or exercising with companion animal: never/at least sometimes; seeking companion animal for comfort: never/at least sometimes), and the change in physical and mental health (outcome variables), adjusting for covariates gender, age, ethnicity, and professional activity.

For all analyses, missing data for covariates were imputed by utilising an imputation model that included all other variables as predictors. This subsequently created 10 imputed datasets that were separately analysed. The results were combined to generate pooled estimates of effects; allowing the analyses to account for uncertainty caused by estimating missing values. Data were analysed using SPSS version 26.0 (IBM^®^). Standard alpha levels were applied in a two-tailed test of significance (*p* < 0.05 considered significant), with family-wise error rate corrected using the false discovery rate [33]. All statistical analyses described above were pre-specified and uploaded on the Open Science Framework (https://osf.io/s5qjb; accessed on 6 June 2021).

In order to address RQ3, free-text responses to the final survey question were uploaded to NVivo 12 software (QSR International Pty Ltd. Version 12, Ottawa, ON, Canada). Thematic analysis was used to analyse the free-text comments [34], adopting an inductive approach, whereby coding and development were driven by response content. The first author read all the free-text responses and generated notes regarding any potential codes by identifying recurrent words and units of meaning. The same author developed initial codes and categorised them into meaningful groups. Subsequently, codes were organised into potential themes and appropriate coded responses were ordered within each identified theme. Lastly, two authors independently reviewed the themes and respective quotations and reached a consensus on the theme assignment.

## 3. Results

A total of 315 participants completed OWLS 1 and consented to complete the follow-up survey. Of those, 66 did not complete OWLS 2, as they were too unwell to participate, they declined to participate, or they were unable to be contacted. This resulted in a final sample of 249 participants. Table 1 presents a summary of participant characteristics.

Just over half of the participants (53.8%, n = 134) were non-animal owners. Of these, 51.5% (n = 69) reported they would like to own an animal, but their current situation would not allow it, 42.5% (n = 57) said they were not interested in owning an animal, and 7.5% (n = 10) indicated they had recently lost an animal and were not ready to have another one yet. Lastly, 20.1% (n = 27) indicated they did not own an animal for an alternative reason. Participants could provide a free-text response for why they did not own an animal for a reason not listed. These frequently included *‘not feeling well enough to own an animal’* and *‘not wanting the responsibility of caring for an animal’.*

### 3.1. Are Well-Being and Loneliness Scores Associated with Animal Ownership in People with SMI during the Pandemic Restrictions? (RQ1)

Adjusting for relevant covariates, companion animal ownership was not significantly associated with total well-being or loneliness scores during the pandemic restrictions (see Table 2). 

### 3.2. Are Deterioration or Improvement in Physical and Mental Health Associated with Animal Ownership and Regular Engagement with Companion Animals? (RQ2)

Adjusting for relevant covariates, animal ownership was significantly associated with self-reported changes in mental health. Animal owners were more likely to report a decline in their mental health over the course of the pandemic. However, there were no significant associations between animal ownership and the self-reported change in physical health. Regular engagement with companion animals, as indicated by the amount of time spent each day playing with or exercising with their animal, or seeking their animal for comfort, were not significantly associated with changes in mental or physical health (see Table 3).

### 3.3. How Do Companion Animal Owners Perceive the Influence of Human–Animal Interaction on Their Physical and Mental Health during the Pandemic Restrictions? (RQ3)

Of 66 participants who provided an optional free-text response, all owned a companion animal. Table 4 presents the full participant characteristics for this sub-sample.

The thematic analysis of free-text responses resulted in the identification of two main themes with related sub-themes, reflecting various aspects of human–animal relationships during the pandemic restrictions (see Table 5). To illustrate themes and sub-themes, the free-text responses are presented as verbatim quotes below, with the gender and age of participants provided in brackets.

#### 3.3.1. Positive Impact of Animal Ownership during COVID-19

##### Amelioration of Mental Health and Well-Being

The majority of participants reflected on the benefits of owning a companion animal for their mental health and well-being. It was frequently expressed that companion animals were able to improve their owners’ mood, reduce their stress, and help owners to cope generally with the COVID-19 restrictions.

*‘My dogs are my best friends, companions and are like my children. In the context of COVID-19, they have helped me to stay calm, focused and happy.’*

(male, 52 years)

*‘I’ve always said that a house isn’t a home without a cat. They bring so much love into the house. Generally, they have been an essential part of my well-being.’*

(female, 52 years)

Participants described positive experiences of how their companion animals had impacted both their past and present mental health status and helped them to cope by providing a continuous source of company and emotional support.

*‘My cat has stopped me from committing suicide many times, her meow brings me out of it.’*

(male, 54 years)

*‘She’s* [dog] *got me through COVID and my addiction—I’m ten years clean. She’s my miracle and my baby. If it wasn’t for her, I don’t think I would be here. She’s been my rock.’*

(male, 45 years)

Consistency, unconditional love, and affection appeared to be central features of narratives about participants’ relationships with their companion animals. A number of participants commented on the non-judgemental nature of the human–animal relationship. Freedom from concerns about judgements allowed participants to be comfortable and open when interacting with their animals, which may have led to a sense of safety and comfort which was not apparent with alternative types of interpersonal relationships.

*‘Pets love you more than they love themselves—their loyalty and love is its own kind! They* [dog and cat] *sit and listen to all my problems without any judgement.’*

(female, 22 years)

*‘The cats pick up on when I’m not feeling well. Stroking my cats helps me to feel loved and comforted. They love me unconditionally. They’re warm and comforting and they never judge. They’re better than people.’*

(female, 77 years)

There was also a consensus that companion animals gave their owners a sense of focus and feelings of purpose during the COVID-19 restrictions, encouraging regularity and consistency to their daily routines. It appeared that providing care to a companion animal facilitated a sense of feeling useful and needed, and this was particularly important during the pandemic context.

*‘Keeping pets* [cat and hamster]* has always helped me maintain a schedule, and feel more useful/needed, which has been more important while being furloughed during the pandemic’*

(female, 26 years)

*‘Having the dogs makes me get up.* [Dog’s name] *is diabetic, so has to be fed at seven, then have his insulin’*

(female, 58 years)

*‘Having the commitment to looking after my cat helps give me purpose’*

(male, 61 years)

One participant expressed that the responsibility of caring for their fish ‘required a lot of upkeep’ but described how it was worth the effort as their fish provided a therapeutic effect in various ways, which subsequently had a positive impact on their well-being.

*‘I find my fish therapeutic. They are generally placid and calm. The sound of the water can have quite a calming effect. They require a lot of upkeep in the sense of regular water changes etc., but they are worth the effort. They can be quite amusing as each of them have their own personality which makes me smile.’*

(male, 42 years)

###### Diminished Sense of Isolation and Loneliness

Many participants shared the sentiment that the constant source of companionship received from their animal was essential during the pandemic restrictions. Owning a companion animal appeared to reduce feelings of loneliness and isolation by providing a sense of ‘connectedness’. Due to the pandemic restrictions, companion animals often fulfilled the primary role in social support networks, particularly for individuals living by themselves.

*‘My pets* [cats] *have been a lifesaver, kept me company through the pandemic. Worked from home a lot more, helped me feel connected when there has been less social contact.’*

(male, 52 years)

*‘Over the pandemic, they* [dogs] *have become my only company. I really can’t imagine how dreadful it would be to have an empty house. They are invaluable to me.’*

(female, 67 years)

Some participants expressed that they derived comfort from the physical contact with their animals which also facilitated a reduced sense of loneliness and isolation.

*‘The dog is why I don’t feel lonely—she stays with me all evening and sits with me. If I stop stroking her then I’m reminded she’s there.’*

(male, 62 years)

*‘I don’t like it if my cat isn’t on my bed at night.’*

(female, 55 years)

A number of participants reported that animal ownership resulted in increased socialisation with a wider social network. This was perceived as particularly beneficial during the social contact restrictions. Lastly, one participant acknowledged that owning a companion animal may facilitate the development of new interpersonal relationships.

*‘My dog is very important to me. He makes me go out daily. I meet other dog walkers and have chats.’*

(female, 53 years)

*‘If I didn’t have my cat, I wouldn’t have anything to talk to. I might think about getting a dog and this might help with other relationships (e.g., getting a partner).’*

(male, 64 years)

###### Physical Health Benefits

Owning a companion animal (primarily dogs) appeared to bring physical health benefits to their owners. Many dog owners reported that owning a dog had promoted exercise and physical activity as they had the responsibility to walk their dog. Subsequently, this led to enhanced mood and well-being due to the promotion of physical activity and time spent outdoors.

*‘It makes me feel better to get outside and walk the dog at least once daily, whatever the weather.’*

(female, 56 years)

*‘The dog gets me outside even when I don’t want to. Even when it’s freezing and raining and I hate the thought of going out, it still gets me up and exercising for an hour.* *’*

(male, 62 years)

###### Increased Appreciation of Animals during COVID-19

Many participants expressed how their animals had helped them to cope in general throughout the pandemic restrictions, and how they had been a *‘huge source of support during this time’* (female, 33 years).

*‘My cats mean the world to me and have made the lockdown easier for me.’*

(male, 40 years)

*‘They are a comfort to both of us and a source of happiness. We are both glad to have our cats especially under lockdown as my wife has mostly had to stay indoors.’*

(male, 63 years)

*‘They* [dog and cat] *have helped so much through COVID and at all times.’*

(female, 52 years)

Some individuals referred to their animals as *‘lifelines’* or *‘lifesavers’.*

*‘Feel like pets* [dogs] *are a lifeline.’*

(female, 59 years)

*‘My dog has been a lifeline during the pandemic. She has given me emotional support.’*

(female, 40 years)

*‘My pets* [cats] *have been a lifesaver, kept me company through the pandemic.’*

(male, 52 years)

#### 3.3.2. Negative Impact and Concerns of Animal Ownership during COVID-19

In general, the free-text comments provided by the participants reported the benefits of owning a companion animal during the pandemic. However, a number of participants also reported negative aspects to animal ownership, highlighting concerns about several features of ownership, such as access to veterinary care and anxieties about their animal experiencing separation-related problems when normal routines resumed.

##### Restricted Access to Veterinary Care

Participants highlighted the difficulties of accessing veterinary care during the restrictions, which exacerbated stress for the owner.

*‘It can be quite tricky if they get sick, it is incredibly hard to give rats medication. In terms of COVID, I struggled to access the medication. I need people to help me administer the medication as sometimes it’s physically impossible to give the medication alone. I would have to break the law by having someone come over to help me, otherwise, the rats would die. This can be quite stressful.’*

(female, 28 years)

However, one participant reported they were able to receive veterinary care at their home as an alternative to visiting the veterinary practice.

*‘Had to have the vet out during lockdown* [for my cat] *(would normally have gone to vet).’*

(female, 69 years)

##### Animals’ Potential Separation-Related Problems

Reported concerns also included the possibility of their animal experiencing separation-related problems upon return to work after an extended period working from home. Participants expressed that their animals had become ‘their only company’ and reported an increase in the time spent together. It was clear that the potential of separation-related problems was exacerbating stress for the owner, as participants stated that they were worried about leaving their home due to the impact this may have on their companion animal.

*‘I fear they now have separation anxiety as a result of us constantly being together.’*

(female, 67 years)

*‘It is stressful to leave them alone now when I need to go out because they have been so used to me being there all of the time.’*

(female, 52 years)

## 4. Discussion

Our survey explored the association between animal ownership in people with SMI and their mental health and physical health, together with their perceptions of human–animal interaction during the COVID-19 pandemic restrictions. To the best of our knowledge, this is the first study to investigate companion animal ownership in people with SMI and its links with mental and physical health during the COVID-19 pandemic. Results from this survey suggest that animal ownership was associated with self-reported deterioration in mental health, contrasting with previous findings in the general population. Despite this association, qualitative data indicated that companion animals did constitute an important source of emotional support to owners during the course of the COVID-19 pandemic, a finding that aligns with existing research in the general population. The discrepancy identified between the qualitative and quantitative findings and the range of established factors influencing the human–animal relationship highlights the complexity of the relationship between an owner and their animal.

### 4.1. Association between Animal Ownership and Mental Health

Results from this survey suggest that well-being and loneliness scores were not significantly associated with animal ownership in people with SMI during the pandemic restrictions. However, having a companion animal was associated with a self-reported decline in mental health, but not physical health, over the course of the pandemic. This is an interesting finding, as it is often assumed that companion animals are beneficial for the mental health of most owners [2,7]. In fact, previous research in the general population has reported that owning a companion animal during COVID-19 pandemic restrictions was associated with less deterioration in mental health [3] and lower depression and anxiety [35,36]. Outside of a pandemic context, one study investigating companion animal ownership and mental health reported the benefits of ownership for military veterans with post-traumatic stress disorder, including reduced feelings of loneliness and depression [37]. There is also evidence indicating the direct effect of animals on depression and mood [37,38], through close physical contact (e.g., grooming and stroking) [38].

It is possible that our findings indicate the self-reported decline in mental health was due to the pandemic restrictions and subsequent challenges of animal ownership that may be amplified during this context. Evidence in the general population suggests that despite an overall consensus of the benefits of animal ownership on mental health and well-being, negative aspects of animal ownership are also frequently identified, which can result in feelings of distress [4,18,20]. For those with diagnosed mental health conditions, the burden of animal ownership (e.g., financial costs and housing situations) can be detrimental to the owner’s well-being [6,38,39,40], and this has been reported outside of a pandemic context. Horses have been considered as the most burdensome in this regard [40], providing a potential explanation for the absence of this species in our sample. Overall, it is plausible to suggest that concerns relating to animal ownership may have been exacerbated by the pandemic restrictions and resulted in a greater self-reported decline in mental health. Further COVID-specific animal ownership concerns (e.g., restricted access to veterinary care or the potential development of separation-related problems, as indicated by the free-text responses) may have also contributed to the self-reported decline in mental health. Therefore, the findings from this study highlight the challenges associated with caring for an animal in a pandemic context, which may intensify owners’ feelings of distress.

Despite the association found between animal ownership and self-reported decline in mental health, it is clear from our thematic analysis that there are potential benefits of animal ownership for people with SMI. This highlights that quantitative and qualitative measures may identify different elements of the relationship between animal ownership and outcomes related to mental health. As indicated by the free-text comments, participants clearly believed animal ownership had resulted in a positive impact for various reasons both within and outside of a pandemic context. Companion animals were perceived to provide a consistent source of emotional support and companionship, a finding which aligns closely with previous research in the general population [4] and also in those with diagnosed mental health conditions [6,8,39]. Therefore, our current findings demonstrate that those with SMI can derive the same benefits from animal ownership as the general population. Animals may play important roles in terms of improving quality of life and well-being considering the amount of social exclusion and stigma that are likely to be experienced by this population [2,6,8,39]. This is likely to be of increasing importance due to social isolation felt by people with SMI which is likely to have been exacerbated by the pandemic restrictions—a cause and effect of mental illness [41]. 

Lastly, evidence in the general population has reported that companion animal owners experience more psychological problems compared to non-owners [42], and strong attachment with animals may predict the mental health vulnerability of the owner [9,10]. These findings align with our quantitative results, which reported that owning a companion animal was significantly associated with a self-reported decline in mental health, potentially indicating mental health vulnerability. However, we did not ask participants to indicate the perceived strength of the bond with their companion animal. This is important as the human–animal bond is a construct that may be connected to mental health vulnerability in companion animal owners [3,9,10]. Further exploration of the owners’ perceived closeness to their companion animal is warranted. Asking animal owners about the perceived strength of their bond to their animal may help to identify potential mental health vulnerabilities and could be beneficial in clinical contexts [3].

We did not identify significant associations between regular engagement with the companion animal and self-reported change in mental or physical health. We are not aware of existing research that has investigated this link but considered evidence of the potential benefits of human–animal interaction in more general terms [43]. It is plausible to assume that engagement with companion animals (e.g., as indicated by the amount of time spent each day playing with or exercising with their animal or seeking their animal for comfort) may result in a human–animal bond that could translate into benefits during the pandemic restrictions. For example, as indicated by our free-text comments, owners derived a great source of emotional comfort from their companion animal and expressed their animals gave them a sense of purpose, focus, and motivation for physical activity. These findings align closely with previous qualitative research investigating the impact of companion animals during the COVID-19 pandemic, which reported similar benefits in a large sample of UK adults [4]. Future research could explore the frequency of engagement with companion animals in more detail, investigating its links with mental health and loneliness. 

### 4.2. Association between Animal Ownership and Physical Health

The existing evidence base indicates that animal ownership may encourage physical activity [44]. This mechanism is strongly supported for companion animal species such as dogs or horses [45,46,47]. Those who own dogs may be more active than those who do not own animals due to the amount of dog walking required [45,46,48], with a well-established connection between exercise and positive mental well-being [49]. This aligns closely with our own qualitative results, which indicated that animal ownership (primarily dogs) encouraged and promoted physical activity, subsequently leading to enhanced mood and well-being. However, we did not identify significant associations between self-reported physical health change and animal ownership in our regression analyses. This is perhaps unsurprising, given only 53% of animal owners in the sample owned dogs, and no participants owned horses or ponies, animals typically associated with promoting physical health and exercise [45,46,47]. The care required and benefits accrued from animal ownership differ based on the companion animal owned, as dogs and horses require more owner-initiated exercise than species such as cats, small mammals, birds, or reptiles [50]—animals commonly owned in the current sample. Therefore, existing evidence provides a more direct link between animal ownership and physical health outcomes among dog and horse owners than among other animal owners [50], similar to our qualitative findings.

### 4.3. Caring for Companion Animals in a Pandemic Context

As indicated by the free-text responses, most animal owners expressed their animals had helped them to cope with the pandemic context and reported deriving a constant source of emotional support from their animals. However, concerns relating to caring adequately for their animals were also reported, similar to previous research with the general population [4,20]. It is essential to understand how COVID-19 specific concerns related to animal ownership may affect certain population groups. This is particularly the case for those with SMI, as concerns related to animal ownership may exacerbate existing feelings of distress or anxiety [4]. Our findings highlight the challenges associated with the responsibility of caring for an animal in a pandemic context and indicate the need to consider the development of additional targeted support approaches for those with SMI in this context. 

### 4.4. Limitations

Firstly, future research would benefit from recruiting a larger sample size and comparing larger groups of animal owners to non-owners. However, a strength of the sample included the number of male participants. It is commonly reported that samples are predominantly female in the field of human–animal interaction research [51], but this gender bias was not present in our sample. Secondly, we were unable to draw out additional themes and sub-themes as we did not conduct interviews with participants. Instead, the responses were collated from an optional survey question. Therefore, it is difficult to ascertain whether data saturation was achieved in a similar way to that which could have been achieved by conducting face-to-face interviews. Due to this, the depth of the data may be restricted compared with an interview approach. Thirdly, the measure we used to collect the frequency of engagement with their companion animal was not standardised. Therefore, a potential effect could have been missed due to instrument weakness. Lastly, we did not collect data related to the perceived strength of the human–animal bond. Previous research has indicated the human–animal bond construct may be connected to mental health vulnerability in those who own animals, and it would have been important to explore this within this population group.

## 5. Conclusions

Our current study provided an in-depth, mixed-method insight into the impact of human–animal relationships for individuals with SMI. Animal ownership in people with SMI was associated with a self-reported deterioration in mental health during the second wave of the pandemic in the UK. However, the findings also highlight the perceived positive impact of animal ownership during this time, as indicated by the free-text responses. Despite these perceived benefits, anxieties relating to caring for animals during this time were also reported. Future qualitative research to unravel the elements contributing to the complex human–animal relationship would be advantageous to facilitate our understanding of the more specific needs of animal owners with SMI. Critically, our findings provide an indication that the well-established understanding that companion animals are beneficial for the mental health of most owners may not be the case for people with SMI in a pandemic context.

## Figures and Tables

**Table 1:** Participant characteristics (n = 249).

Characteristics		% (N)
Gender	Female	46.6 (116)
	Male	51.4 (128)
	Transgender	2.0 (5)
Age (years)	18–24	3.6 (9)
	25–34	10.0 (25)
	35–44	20.1 (50)
	45–54	22.1 (55)
	55–64	19.7 (49)
	65–70	12.0 (30)
	Over 70	12.5 (31)
Ethnicity	White	84.3 (210)
	Other ethnic	15.7 (39)
Professional activity	Professionally active	35.3 (88)
	Not professionally active	64.7 (161)
Companion animal ownership	Yes	46.2 (115)
Companion animal species	Dogs	53.0 (61)
	Cats	47.8 (55)
	Small mammals	11.3 (13)
	Fish	8.7 (10)
	Horses or ponies	0 (0)
	Birds	1.7 (2)
	Reptiles or amphibians	4.3 (5)
	Farm animals	2.7 (2)
	Other	2.6 (3)

**Table 2:** Linear regression models of association of animal ownership and well-being and loneliness scores, adjusting for relevant covariates.

Predictor	Total Well-Being Score			
	***b*** **_adj_**	**95% CI**	***p*** **-value**	**R^2^**
**Animal ownership ^1^**	−0.633	−2.903–1.637	0.583	0.050
	**Total loneliness score**			
**Animal ownership ^2^**	0.039	−0.539–0.617	0.893	0.021

^1^ Gender*, age, ethnicity, professional activity; ^2^ gender, age, ethnicity, professional activity; * indicates significance (*p* < 0.05).

**Table 3:** Binary logistic regression models of association of predictors and self-reported change in mental and physical health, adjusting for relevant covariates.

Predictor	Mental Health Change			
	*b*_adj_	95% CI	*p*-value	R^2^
Animal ownership ^1^	0.640	0.102–1.231	0.025 *	0.040
Time spent with companion animal ^2^	−0.540	−1.717–1.381	0.501	0.025
Seeking companion animal for comfort ^3^	−0.346	−2.311–01.534	0.593	0.026
	Total Loneliness Score			
	*b*_adj_	95% CI	*p*-value	R^2^
Animal ownership ^1^	−0.014	−0.537–0.562	0.961	0.016
Time spent with companion animal ^2^	0.466	−1.622–1.159	0.519	0.050
Seeking companion animal for comfort ^3^	−0.981	−2.037–0.962	0.219	0.067

^1^ Gender, age, ethnicity, professional activity; ^2^ gender, age, ethnicity, professional activity; ^3^ gender, age, ethnicity, professional activity; * indicates significance (*p* < 0.05).

**Table 4:** Participant characteristics for sub-sample who provided a response to the optional free-text item (n = 66).

Characteristics		% (N)
Gender	Female	51.5 (34)
	Male	45.5 (30)
	Transgender	3.0 (2)
Age (years)	18–24	4.5 (3)
	25–34	9.1 (6)
	35–44	10.6 (7)
	45–54	22.7 (15)
	55–64	25.8 (17)
	65–70	18.2 (12)
	Over 70	9.1 (6)
Ethnicity	White	90.9 (60)
	Other ethnic	9.1 (6)
Professional activity	Professionally active	40.9 (27)
	Not professionally active	59.1 (39)
Companion animal ownership	Yes	100 (66)
Companion animal species	Dogs	54.5 (36)
	Cats	43.9 (29)
	Small mammals	9.1 (6)
	Fish	12.1 (8)
	Horses or ponies	0 (0)
	Birds	0 (0)
	Reptiles or amphibians	4.5 (3)
	Farm animals	3.0 (2)

**Table 5:** Themes and related sub-themes.

**Theme One: Positive impact of animal ownership during COVID-19** Amelioration of mental health and well-being Diminished sense of isolation and loneliness Physical health benefits Increased appreciation of animals during COVID-19
**Theme two: Negative impact and concerns of animal ownership during COVID-19** Restricted access to veterinary care Animals’ potential separation-related problems

## Data Availability

We are not able to share a de-identified dataset as we do not have consent from the research participants to do this. We have checked with the GDPR team at the University of York, and they have advised us that we cannot upload these data to a public repository without explicit consent from the study participants. Data requests for the full dataset may be sent to the Closing the Gap Network email: ctg-network@york.ac.uk whose Steering Committee manages our data access requests.

## Supplementary Materials

The following are available online at https://www.mdpi.com/article/10.3390/ijerph182211908/s1, Supplementary Material S1: Optimising Well-being in Self Isolation Study: Methodology.
