# Supplementary material for: The Role of Animal Ownership for People with Severe Mental Illness during the COVID-19 Pandemic: A Mixed-Method Study Investigating Links with Health and Loneliness
Source: Int J Environ Res Public Health. 2021 Nov 12;18(22):11908. doi: 10.3390/ijerph182211908 (PMC8624942; doi:10.3390/ijerph182211908)

Supplementary Material 1.

Optimising Wellbeing in Self Isolation study: Methodology

Design and Procedures

This study reports on results obtained from the Optimising Wellbeing in Self-Isolation study – phase 2 (OWLS 2; data collected from January 2021 to March 2021) which drew participants from OWLS – phase 1 (OWLS 1; data collected from July 2020 to December 2020). OWLS 1 participants were a sub-group of participants from the Closing the Gap (CtG) study (data collected from April 2016 to May 2020).

The CtG study was a large clinical cohort (N = 9,914) comprising adults (aged 18 years or older) with documented diagnosis of schizophrenia or delusional/psychotic illness (ICD 10 F20.X & F22.X or DSM equivalent) or bipolar disorder (ICD F31.X or DSM equivalent). Ethical approval for the CtG study was granted by West Midlands—Edgbaston Research Ethics Committee (REF 15/WM/0444).

OWLS 1, recruited a sub-cohort from CTG, to explore the effects of the COVID-19 pandemic restrictions on people with severe mental ill health. To be eligible for invitation to OWLS, CtG participants had to have provided contact details and consented to be contacted again, as well as been originally recruited from a clinical site that had the capacity to collaborate with the University of York research team in a new research project. Eligible participants were then organised in groups based on age, gender, ethnicity, and care setting (primary or secondary mental health care) to ensure representation across many sociodemographic groups. From each group, researchers selected a purposive sample of participants that had most recently participated in the CtG study (e.g., recruited in the last two years) ensuring that a range of localities was covered. Recent participation to the CtG was considered important to increase response rates (e.g., the team having current and valid contact details, and participants being familiar with the research team). Locality was used to provide geographical diversity, inviting participants from 17 mental health trusts and six Clinical Research Network (CRN) areas in England, including a mix of rural and urban settings.

Participants who consented to follow up in OWLS 1 were invited to take part in OWLS 2. In both studies, participants who expressed an interest in taking part were provided with an information sheet (read over the phone, or send by email, text message, or post). Those consenting to participate were given the option to complete the survey over the phone with a researcher, online, or completing and returning a hard copy survey sent by post. Ethical approval for the OWLS project was granted by the Health Research Authority Northwest – Liverpool Central Research Ethics Committee (REC reference 20/NW/0276).

Samples

Out of 2,932 participants in the CtG study that were eligible to be invited to OWLS 1, we selected a purposive sub-sample of 1,166 (39.8 %) participants and successfully contacted 688 (59%). The survey was completed by 367 participants (31.5% of the selected sub-sample and 53.3% of those successfully contacted). The final study sample had a mean age of 50.5

(± 15.69) years old and it included 51.0% men, 47.4% women, 1.6% transgender, 17.7% people from other than White ethnic background and 48.5% residing in high/very high deprivation areas in the country. The primary diagnosis was psychosis (51.2%). The survey was completed online by 121 participants (33%) and over the phone or via the post by 246 (67%). In terms of location, 51.2% of participants were recruited from the North of England, 5.7% from East Midlands/Anglia, 10.4% from London, and 32.7% from the South of England. Out of 367 participants in the OWLS 1, 315 consented to follow up and 249 participated in OWLS 2 (79.0%). Participants in OWLS 2 did not differ from OWLS 1 participants that did not participated in OWLS 2, in any sociodemographic characteristics (Age: $t(365) = -0.45$, $p = .650$; Gender: Likelihood Ratio (2) = 4.77, $p = .092$; Ethnicity: $\chi2$ (1) = 1.44, $p = .230$; Deprivation: $\chi2(4) = 6.47$, $p = .167$; Care setting: $\chi2(1) = 0.63$, $p = .429$; Diagnosis: $\chi2(3) = 6.07$, $p = .108$). OWLS 2 participants had a mean age of 51.7 years old (range: 21-84) and the sample included 51.4% men, 6.6% women, 2% transgender, 15.6% people from other than White ethnic background, and 44.6% residing in high/very high deprivation areas in the country. The primary diagnosis was psychosis-spectrum disorder (48.2%). The survey was completed online by 93 participants (37.3%) and over the phone or via the post by 156 (62.7%). In terms of location, 48.9% of participants were recruited from the North of England, 4.8% from East Midlands/Anglia, 9.2% from London, and 37.0% from the South of England.

The OWLS surveys

Participants in OWLS 1 self-reported information on the following domains: 1. Mental and physical health and wellbeing, 2. Experiences using healthcare services, 3. Health-related behaviours (e.g., smoking, alcohol, diet, physical activity, etc.), 4. COVID-19 specific experiences (e.g., being ill with COVID-19, having to self-isolate, etc.), 5. Loneliness and social support, 6. Use of the Internet and digital devices, and 7. Employment and financial status.

Participants in OWLS 2 self-reported information on the same domains as in OWLS 1, plus a domain about the role of pets and animal during the pandemic. Although items in the two surveys overlap to some extent, OWLS 2 also included additional or amended items to further explore some of the issue identified in OWLS 1.

Surveys are available in the OWLS repository in the Open Science Framework.
OWLS1:
https://mfr.osf.io/render?url=https://osf.io/qpfn6/?direct%26mode=render%26action=download%26mode=render f
OWLS2:
https://mfr.osf.io/render?url=https://osf.io/7wuae/?direct%26mode=render%26action=download%26mode=render

Figure S1: Flow Diagram - OWLS

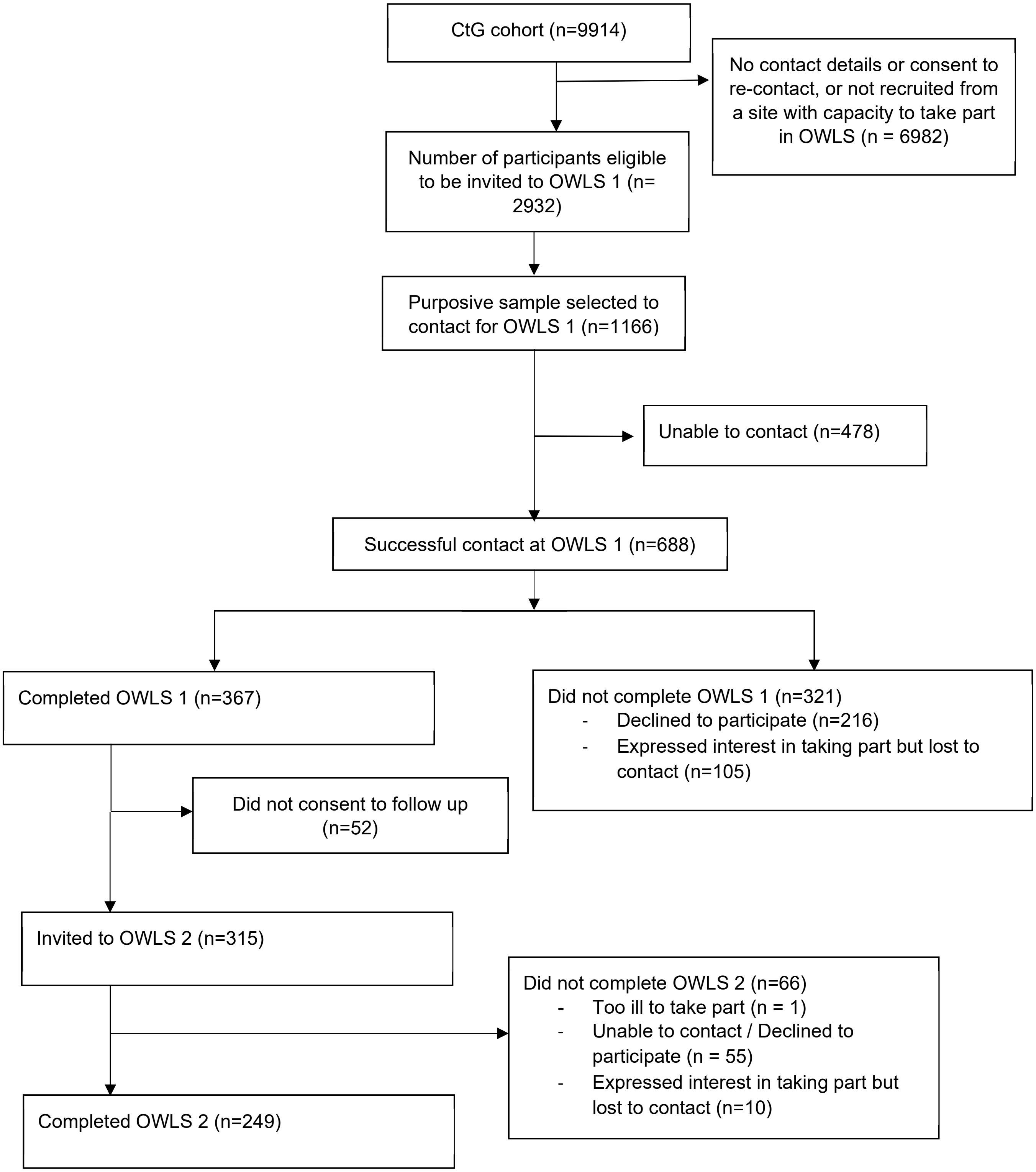

Supplement: Supplementary file 1 [file ijerph-18-11908-s001.zip › ijerph-1437141-supplementary.pdf]
